# Vitamin D_3_: A promising antifungal and antibiofilm agent against *Candida* species

**DOI:** 10.18502/cmm.2023.345062.1416

**Published:** 2023-06

**Authors:** Zahra Kherad, Somayeh Yazdanpanah, Farshid Saadat, Keyvan Pakshir, Kamiar Zomorodian

**Affiliations:** 1 Department of Medical Mycology and Parasitology, School of Medicine, Shiraz University of Medical Sciences, Shiraz, Iran; 2 Department of Immunology, Faculty of Medicine, Guilan University of Medical Sciences, Rasht, Iran; 3 Center of Basic Sciences in Infectious Diseases, School of Medicine, Shiraz University of Medical Sciences, Shiraz, Iran

**Keywords:** Antifungal agent, Biofilm, Candidiasis, *Candida albicans*, Vitamin D_3_

## Abstract

**Background and Purpose:**

*Candida* species are opportunistic fungal pathogens that cause mild to life-threatening infections in both immunocompetent and immunocompromised populations. The increasing prevalence of drug-resistant *Candida* species has posed a significant challenge to the management of infections in clinical settings. Therefore, this study aimed to investigate the direct antifungal and antibiofilm effect of vitamin D_3_ against *Candida* species.

**Materials and Methods:**

The antifungal activity of vitamin D_3_ was evaluated by broth microdilution method based on the Clinical and Laboratory Standard Institute. Prevention of biofilm formation by *Candida albicans* was measured using the XTT assay following exposure to different concentrations of vitamin D_3_. Moreover, expression of *Agglutinin-like sequence* gene 1 (*ALS*1), *hyphal wall protein* gene (*HWP*1), *secreted aspartyl proteinase* 6 gene (*SAP*6), and *morphogenesis pathway regulatory* gene (*EFG*1) were analyzed by real-time polymerase chain reaction using the comparative Ct method (ΔΔ Ct) after exposure to vitamin D_3_.

**Results:**

Vitamin D_3_ showed antifungal activity against *Candida* species ranging from 1-128 μg/mL. Furthermore, vitamin D_3_ inhibited biofilm formation in a dose-dependent manner, with IC50 of 7.5 μg/mL. Treatment with vitamin D_3_ resulted in significant upregulation of the *EFG*1, *ALS*1, and *SAP*6 genes under hypha-inducing conditions to overcome environmental challenges.

**Conclusion:**

Results of the current study demonstrated that vitamin D_3_ has a significant inhibitory effect on *Candida* growth and biofilm formation. Considering its demonstrated antifungal and antibiofilm properties, vitamin D_3_ holds promise as a potential agent for medical applications.

## Introduction

Candida species are members of the human normal mucosal flora that cause invasive lethal infections, particularly candidemia, in immunocompromised people [ 1 ]. Under certain environmental and nutritional conditions, this unicellular yeast changes to a hyphal form with the ability to invade tissues. The complicated structure of yeasts and hyphae with an extracellular matrix, called biofilm, is attributed to pathogenicity and the exhibition of high resistance to antifungal drugs [ 2 ]. Therefore, studies on the mechanism of biofilm formation and the anti-biofilm activity of various components have increased considerably.

Biofilm development is initiated by the expression of some genes, including *hyphal wall protein* gene (*HWP*1) and *Agglutinin-like sequence* (*ALS*) family genes that produce adhesin molecules to facilitate the adherence of *C. albicans* to other cells or surfaces [ 3 - 5 ]. In addition, *secreted aspartyl proteinase* (*SAP*), genes are responsible for encoding aspartyl proteinase enzymes that are of considerable importance in the pathogenicity process [ 6 ]. Morphogenesis in *C. albicans* is controlled by regulatory genes, such as *morphogenesis pathway regulatory* gene (*EFG*1), a transcriptional factor that influences the expression of other genes. Hence, the morphology of yeast cells in different environments is determined by the level of *EFG*1 activity [ 7 ].

There is a growing need to introduce new antifungal agents to deal with the emergence of resistant *Candida* species or infections related to biofilm formation. An effective strategy to develop such agents may involve investigating the activity of available compounds. In this regard, vitamin D_3_ is an important mineral associated with the hemostasis of calcium whose insufficiency is related to the risk of several disorders, such as osteoporosis, diabetes, and hypertension [ 8 ]. Importantly, scientific reports have emphasized the significant role of vitamin D_3_ in the immune system and inflammatory processes [ 9 ]. Besides, lower levels of vitamin D_3_ have been associated with bacterial and viral infections, such as tuberculosis [ 10 ]. 

Recently, increasing publications suggest that low levels of vitamin D_3_ are associated with an increased risk of COVID-19 infection and poor outcomes [ 11 , 12 ], indicating a new era for studying the impact of vitamin D_3_ in the prevention and control of infectious diseases. However, there is a limitation of studies on the antifungal activity of vitamin D_3_. Hence, this research aimed to determine the antifungal properties of vitamin D_3_ against *Candida* species, and also investigate its effect on biofilm formation and the expression of certain genes involved in the morphogenesis and pathogenicity of *C. albicans*.

## Materials and Methods

### Antifungal activity (minimum inhibitory concentrations and minimum fungicidal concentrations)

The antifungal activity of vitamin D_3_ was determined against standard strains of *Candida* species, including *C. albicans* (ATCC 10261), *C. tropicalis* (ATCC 750), *C. krusei* (ATCC 6258), *C. glabrata* (ATCC 90030), *C. dubliniensis* (CBS 8501), and *C. parapsilosis* (ATCC 4344).

The minimum inhibitory concentrations (MIC) and minimum fungicidal concentrations (MFC) of vitamin D_3_ against standard species of *Candida* were determined by the broth microdilution method as recommended by the Clinical and Laboratory Standard Institute, with some modifications [ 13 ]. Briefly, the RPMI-1640 (with L-glutamine and phenol red, without bicarbonate) (Sigma, USA) was prepared and buffered with morpholino propane sulfonic acid (Sigma-Aldrich, Stein Heim, Germany) at 165 mM and pH 7 [ 14 ]. Two-fold serial dilution of the vitamin D_3_ (Caspian pharma; Lot No: UE 01406003; Potency: 99.95) ranging from 0.05 to 512 μg/mL were prepared in 96-well microtiter plates using RPMI-1640 media. Yeast strains were sub-cultured on Sabouraud dextrose agar (SDA) (HiMedia Laboratories, India) and incubated at 32-35° C for 12-18 h.

Stock inoculums were prepared by suspending pure colonies of the yeasts in 5 mL sterile 0.85% NaCl and adjusting the turbidity of the inoculums to 0.5 McFarland standard at 530 nm wavelength (1–5 × 10^6^ cells/mL). The working suspension was prepared by making a 1/1,000 dilution with RPMI-1640 of the stock suspension. Therefore, the final concentration of *Candida* inoculum density in each well was 0.5-2.5×10^3^ cells/mL. Following the addition of 0.1 mL of the working suspension to each well, the trays were incubated at 32 °C for 24-48 h in a humid atmosphere. Growth controls (medium without vitamin D_3_) were also included in each row of plates. The MICs were visually determined and defined as the lowest concentration of vitamin D_3_ that inhibited the growth of yeasts. Each experiment was performed in triplicate. The MFCs of vitamin D_3_ were also determined by transferring 10 μL of wells onto SDA plates. The lowest concentration of vitamin D_3_ in which fungal growth was prevented, which corresponded to 98% killing activity, was referred to as the MFC value.

### Biofilm prevention

Inhibition of *C. albicans* biofilm following exposure to vitamin D_3_ was performed as previously described [ 15 ]. A loopful of *C. albicans* (ATCC 10261) colonies was transferred into 25 ml of Sabouraud Dextrose Broth medium (HiMedia Laboratories, India), and incubated in a shaking incubator at 32 ºC for 18 h. After incubation time, the yeast cells were collected by centrifugation and washed twice with sterile phosphate-buffered saline (PBS). Washed cells were added to RPMI-1640 medium, and the absorbance at 530 nm using a spectrophotometer was measured to adjust the cell density corresponding to 1×10^6^ cells/ml. To assay for biofilm prevention, the prepared cell suspension in RPMI-1640 medium was added to wells of 96-well flat-bottomed polystyrene microtiter plates. After incubation at 37 °C for 2 h, wells were gently washed twice with 1X PBS to remove non-adherent cells. Afterward, a fresh medium supplemented with serial dilution of vitamin D_3_ at a concentration of 1.78-60 μg/mL was added. The plates were incubated at 32-35 °C for 48 h. Cell suspension without the addition of vitamin D_3_ was considered the growth control, while wells containing RPMI-1640 alone were considered the negative control.

Biofilm cell metabolic activity was measured using the XTT reduction assay according to a previously reported method [ 16 ]. The XTT (2,3-bis-(2-methoxy-4-nitro-5-sulfophenyl)-2H-tetrazolium-5-carboxanilide) is a tetrazolium salt which converts to colored-product formazan by the activity of mitochondrial dehydrogenase in viable cells [ 17 ]. The supernatant of the wells was gently aspirated at the end of the incubation, and the wells were washed twice with PBS. The XTT solution was prepared by dissolving XTT powder (Sigma) in PBS, and 100 µL of XTT solution containing 4 mM of menadione (Merck, 10 mM in acetone) was immediately added to the wells. Plates were incubated in the dark for 2 h at 37 °C. Afterward, 100 µL of colored supernatant was transferred to a new plate. The optical density (OD) of the supernatant was measured at 490 nm using an automated microplate reader (POLARstar Omega). All assays were performed in triplicate.

### Effect of Vitamin D_3_ on Gene Expression in Candida albicans by real-time polymerase chain reaction

A cell suspension of *C. albicans* equal to 6×10^8^ cells/ml was prepared. After incubation at 37 ˚C for 24 h, the supernatant was removed and the cell pellet was washed twice with PBS. *Candida albicans* was cultured in RPMI-1640 medium with either different concentrations of vitamin D_3_ (6, 60 μg/ml) or fluconazole (2 mg/ml), or with none of these materials (control). After incubation at 37 ˚C for 5 h, cell pellets were collected by centrifugation.

The RNA extraction was performed using the RNeasy Mini Kit (QIAGene) according to the protocol of the manufacturer with some modifications. Moreover, the RNase Free DNase set (QIAGene) was used to preserve the extracted mRNA from contamination. Extracted RNA was used to synthesize cDNA using a kit (Cinna Gene Co.) and followed by amplification with SYBR green real-time polymerase chain reaction (RT-PCR) master mix in a final volume of 20 µL. The RT-PCR primers related to the morphogenesis and pathogenesis of *C. albicans* were applied, including hypha-specific genes (*ALS1* and *HWP1*), *secreted aspartyl proteinase* gene (*SAP6*), and *morphogenesis pathway regulatory* gene (*EFG1*). The RT-PCR experiments were performed using the StepOnePlus^TM^ Real-Time PCR System (Applied Biosystems). The temperature conditions for tests were set to 10 min at 95 ˚C, followed by 45 cycles of 15 s at 95 ˚C, and 1 min at 60 ˚C, with each reaction also performed in triplicate. 

At the end of each reaction, amplification plots and melting temperature curves were analyzed to determine the accuracy of amplifications. Each primer pair produced a single peak in melting temperature curves which demonstrated their specificity. The *ACT1* was selected as a housekeeping gene for normalization of gene expression [ 18 ]. The relative fold changes in gene expression levels were calculated using the comparative Ct method (ΔΔ Ct). Primer sequences are shown in Table 1.

**Table 1:** Primer sequences used for the quantitative real-time-polymerase chain reaction.

Gene	Primer sequence (5´- 3´)	Amplicon size (bp)
*ACT1(Actin 1)*	F: GCTGGTAGAGACTTGACCAACCA	**87**
	R: GACAATTTCTCTTTCAGCACTAGTAGTGA	
*ALS1 (Agglutinin-like sequence 1)*	F: CCTATCTGACTAAGACTGCACC	**184**
	R: ACAGTTGGATTTGGCAGTGGA	
*Hwp1 (Hyphal Wall Protein 1)*	F: CTCCAGCCACTGAAACCACCA	**67**
	R: GGTGGAATGGAAGCTTCTGGA	
*EFG1(Morphogenesis Pathway Regulatory 1)*	F: TATGCCCCAGCAAACAACTG	**202**
	R: TTGTTGTCCTGCTGTCTGTC	
*SAP6 (Secreted Aspartyl Proteinase 6)*	F: TTACGCAAAAGGTAACTTGTATC AAGA	**102**
	R: CCTTTATGAGCACTAGTAGACCAAACG	

### Statistical analysis

All experiments were performed in triplicate for accuracy and reproducibility of tests. Statistical significance of differences between the control group and treated cells with different concentrations of vitamin D_3_ were determined by one-way analysis of variance. The *P* values of less than 0.05 were considered statistically significant. The data were analyzed using the SPSS statistical package (version 16). 

## Results

### Antifungal activity

The results indicated that vitamin D_3_ showed antifungal activity against all tested yeasts. The minimum inhibitory concentrations of vitamin D_3_ against *Candida* species varied from 1 to 128 μg/mL as shown in Table 2. Furthermore, vitamin D_3_ exhibited fungicidal activity (MFC) for all examined yeasts at concentrations ranging from 4 to ˃512 μg/mL. Notably, the highest MIC value was observed for *C. dubliniensis*, while the lowest MIC value was recorded for *C. parapsilosis*.

**Table 2:** Antifungal activity (MICs and MFCs) of vitamin D_3_ against standard *Candida* Species.

*Candida* Species	Fluconazole	Vitamin D_3_	
	MIC (µg/mL)	MIC (µg/mL)	MFC (µg/mL)
*Candida albicans* (ATCC 10261)	4	32	**512**
*Candida dubliniensis* (CBS 8501)	2	128	**>512**
*Candida tropicalis* (ATCC 750)	32	16	512
*Candida parapsilosis* (ATCC 4344)	2	1	4
*Candida glabrata* (ATCC 90030)	32	4	512
*Candida krusei* (ATCC 6258)	64	16	64

MIC: Minimum Inhibitory Concentration, MFC: Minimum Fungicidal Concentration

### Inhibition of Biofilm Formation

According to the results, vitamin D_3_ showed significant efficacy against *C. albicans* biofilm formation, reducing biofilm formation up to 88% at a concentration of 60 µg/mL, as shown in Table 3. The antibiofilm activity of vitamin D_3_ was found to be dose-dependent, with an IC_50_ value of 7.5 µg/mL.

**Table 3:** Quantitative measurement of the biofilm formation of *Candida albicans* in exposure to different concentrations of vitamin D_3_.

Vitamin D_3_ Concentration (µg/mL)	Od (490 nm)	Dead Cells (%)
60	0.1	88%
30	0.29	68%
15	0.38	60%
7.5	0.45	52%
3.75	0.5	47%
1.78	0.6	36%

### Effect of vitamin D_3_ on gene expression

To identify the relationship between vitamin D_3_ and the morphogenesis/pathogenesis of *C. albicans*, quantitative RT-PCR was performed to assess the expression of cell surface protein genes (*ALS1* and *HWP1*), secreted aspartyl proteinase (*SAP6*), and regulatory gene (*EFG1*) (Table 4). 

**Table 4:** Fold changes in gene expression under hyphae-inducing condition (RPMI-1640 medium, incubation at 37 °C for 6 h) using the comparative Ct method (ΔΔ Ct).

Gene	Untreated *Candida albicans*	Fluconazole (2 µg/mL)	Vitamin D_3_	
			6 μg/mL	60 μg/ml
***ALS1***	0.98	0.56	2.61**	11.96**
***SAP6***	1.0	0.60	3.17**	3.27**
***HWP1***	1.0	0.15	0.93	1.20
***EFG1***	1.0	0.46	12.48**	11.21**

* P-value ≤ 0.001: *P*-values were obtained after the comparison of the test results with untreated *C. albicans*.

Transcription of genes was quantified by reverse transcription-quantitative polymerase chain reaction. Samples were treated with Vitamin D_3_ under hyphae-inducing conditions for RNA extraction. Analysis was carried out by analysis of variance. Fold change was calculated by PCR product of the gene of interest/the PCR product of *ACT1* (the housekeeping gene) and normalized to the negative control of untreated *C. albicans* where the expression was considered equal to 1.

Exposure of *C. albicans* cells to vitamin D_3_ significantly changed the expression of an important gene involved in the hyphal growth of *C. albicans*. According to the results, remarkable upregulation of the *EFG1* gene, a transcription factor, was observed following the exposure of *C. albicans* to both low and high concentrations of vitamin D_3_. The *SAP6*, a biofilm-specific aspartyl protease gene, was upregulated nearly threefold in vitamin D_3_-treated *C. albicans* cells versus untreated cells in both low and high concentrations. Moreover, the expression of the ALS1 gene underwent an approximately 12-fold increase at a higher concentration of vitamin D_3_. In comparison with untreated cells, there were no considerable changes in the expression of the HWP1 gene following the exposure of yeast cells to different concentrations of vitamin D_3_. Under hypha-inducing conditions, expression levels of *EFG1*, *ALS1*, and *SAP6* were significantly upregulated after treatment with vitamin D_3_.

## Discussion

The results showed that vitamin D_3_ possesses a potential antifungal activity against *Candida* species, as well as inhibiting biofilm formation. In addition, the expression of genes related to morphogenesis and biofilm formation, including *ALS1*, *SAP6*, and *EFG1*, were significantly upregulated following exposure of the *C. albicans* to vitamin D_3_.

A previous study conducted by Bouzid et al. reported that vitamin D_3_ exhibited inhibitory effects on the growth of *C. albicans* in concentrations of 1.58±0.0764 μg/mL [ 19 ]. Additionally, another study found that the average MIC of vitamin D_3_ against both standard and clinical isolates of *Candida* species was 0.4 mg/mL [ 20 ]. Our findings aligned with these previous studies, which have consistently demonstrated the strong antifungal activity of vitamin D_3_ against *Candida* species. Besides, previous research has also shown that vitamin D_3_ has a bactericidal effect against *Helicobacter pylori* and *Streptococcus mutans* [ 21 , 22 ]. 

The mechanism of action of vitamin D_3_ can be explained by its steroidal properties. Hence, the anti-fungal activity of vitamin D_3_ may be due to its lipid solubility which results in changes in cell membrane integrity and the death of fungal cells [ 23 ]. In addition, steroidal substances have a direct biological effect on the cellular steroids of eukaryotes, including those containing ergosterol. As a result, the antifungal activity of vitamin D_3_ could be explained by its fat solubility [ 24 ]. Furthermore, steroidal substances have direct biological effects on the cellular steroids of eukaryotes, including those containing ergosterol. Therefore, vitamin D_3_ could be considered an antifungal agent due to its inhibitory activity against yeast cells and also its fungicidal activity.

Certainly, the majority of *Candida*-related infections are associated with biofilm formation on surfaces [ 25 ]. Based on the results, vitamin D_3_ significantly inhibited biofilm formation in *C. albicans*. Our data are in agreement with those of an experiment performed by Lei et al., who demonstrated the antifungal effect of vitamin D_3_ on the development and maturation phases of biofilm formation in *C. albicans* [ 20 ]. The potent inhibitory activity of vitamin D_3_ on biofilm formation by *C. albicans* suggests that this micronutrient can be extremely useful in preventing *Candida* biofilm growth. Since *Candida* biofilm is resistant to antifungal drugs due to its specific structure [ 2 , 25 ], vitamin D_3_ can be used for the prevention of *Candida* biofilm formation.

Several studies have reported the upregulation of virulence genes in *C. albicans* in response to antifungal drugs or environmental stresses. For instance, it has been demonstrated that exposure of *C. albicans* to fluconazole results in the upregulation of genes involved in drug efflux pumps (CDR1 and CDR2) and stress response (HSP90) [ 26 ]. In another study conducted by Silva et al. (2017), it was found that exposure to environmental stressors, such as oxidative stress or nutrient limitation, induced the upregulation of genes associated with filamentation (*EFG1* and *HWP1*) and adhesion (*ALS3* and *HWP1*) in *C. albicans* [ 27 ]. 

Similarly, findings of the present study revealed that after exposure to vitamin D_3_, *C. albicans* cells showed a significant upregulation of the *EFG1* gene which required hyphal growth [ 7 ]. Although the treatment showed no remarkable alteration in the expression of *HWP1*, the level of ALS1 gene expression increased which may facilitate biofilm formation. In a previous study, *ALS3* and *HWP1* downregulated after treatment with 0.4 mg/mL of vitamin D_3_ which is in contrast with the findings of the present study [ 20 ]. These variations in gene expression could be explained by the substantially higher vitamin D_3_ concentration that was employed as an antifungal agent in this study. These results imply that *C. albicans* experiences adaptive responses by upregulating virulence-related genes to resist antifungal therapy and environmental challenges. These adaptive responses may help to explain why the genes *ALS1*, *SAP6*, and *EFG1* were upregulated in this investigation.

Studies on the expression of more genes related to pathogenicity will undoubtedly provide a better perspective for mechanisms underlying the effects of vitamin D_3_, which were not possible due to the limited budget and resources of this research. Although the current study showed the remarkable antifungal activity of vitamin D_3_, it is strongly advised to examine the *in vivo* investigations and morphological changes of fungal cells after exposure to vitamin D_3_ using a scanning electron microscope to strengthen and boost the validity of the study.

## Conclusion

Collectively, this study demonstrated that vitamin D_3_ had a significant inhibitory effect on the growth of *Candida* species. Moreover, vitamin D_3_ revealed an intriguing property by inhibiting the biofilm formation in *C. albicans*. In addition, the expression of *ALS1*, *SAP6*, and *EFG1* genes increased following the treatment of yeasts with vitamin D_3_ which represents the adaptive response of *C. albicans* to counteract the inhibitory effects of vitamin D_3_. It is plausible that the upregulation of these genes might contribute to the survival mechanisms of the yeast cells under stressful conditions induced by vitamin D_3_ exposure. 

In general, it appears that vitamin D_3_ can be used as an adjacent therapy for candidiasis since it has inhibitory effects on the growth and development of *Candida* biofilm. However, additional *in vivo* research is still required to establish this antifungal activity.
